# Community-based interventions to reduce dental caries among 24-month old children: a pilot study of a field trial

**DOI:** 10.1186/s12903-021-01999-x

**Published:** 2021-12-10

**Authors:** Marzie Deghatipour, Zahra Ghorbani, Amir Hossein Mokhlesi, Shahla Ghanbari, Mahshid Namdari

**Affiliations:** 1 Department of Community Oral Health, Dental School, Shahid Beheshti University of Medical Sciences, Tehran, Iran; 2 Present Address: Dental Research Center, Research Institute of Dental Sciences, Dental School, Shahid Beheshti University of Medical Sciences, Tehran, Iran; 3 Deputy for Health Affairs, Shahid Beheshti University of Medical Sciences, Tehran, Iran

**Keywords:** Dental caries, Early childhood caries, Community-based interventions, Children

## Abstract

**Background:**

Early childhood caries (ECC) is the most common dental disease among children worldwide, leading to many difficulties on child’s growth. As WHO mentioned, educational interventions in addition to interprofessional collaboration are needed to achieve proper ECC prevention. In present study we’ve aimed to evaluate the effectiveness of some oral health promotion interventions to reduce dental caries among 24-month old children.

**Methods:**

A field trial study was conducted amongst 439 mothers from pregnancy up to 24 months after delivery in Public Health Centers in Varamin, Tehran, Iran. Participants were allocated to intervention (n = 239) and control groups (n = 200). Demographic, socioeconomic status and dental care behavior data were collected using a questionnaire. The content of our study intervention consisted of nutritional and behavioral oral health-related messages. Mothers received messages via either of four methods (A: comprehensive method including all other methods together (n = 74), B: group discussion by dentists (n = 59), C: face to face education by primary health care providers (n = 53), and D: social network (n = 53). The control group received routine maternal and oral health care. To assess the effectiveness of interventions on promoting children’s oral health, the oral health-related behaviors data, the number of decayed teeth (d), and being caries free at the age of two were considered.

**Results:**

Among the 436 examined children, with a mean age of 23.7 months, 48.2% were male. The frequency of using finger toothbrush increased from 53.4% to 89.8% in all intervention groups. The mean (SD) of decayed teeth at 24 months in intervention and control group were 0.36 (0.93) and 1.61 (2.61), respectively. All the four intervention groups, except social network, had more chance of being caries free compared to control group (*P* value < 0.05). Analysis showed that children in comprehensive intervention group had a higher chance of being caries free compared to all other groups, after adjustment for covariates.

**Conclusions:**

Performing oral health interventions could help the prevention of dental caries in newborn children. Also, using a combination of different methods of sending messages can have the best results in promoting oral health.

## Background

Dental caries is one of the most common prevalent global chronic diseases in childhood. It is associated with some adverse health problems for infants and also burdens their families financially [1, 2]. Early Childhood Caries (ECC) is a special kind of dental caries which occurs in primary dentition of young children and it is defined as presence of one or more decayed (non-cavitated or cavitated), missed as a result of caries, or filled tooth surfaces in any primary tooth in a child with 71 months of age or younger [3]. ECC is well-known to be a multifactorial disease with numerous environmental, biological, nutritional and behavioral risk factors such as low parental education level, childhood poverty, inappropriate life style infant feeding practices, daily consumption of sugary drinks, cariogenic diet, poor oral hygiene, lack of professional dental care advice, less dental visits of parents and their children [4–6]. ECC can greatly affect a person's quality of life due to pain, discomfort, functional limitations and social isolation [7, 8]. Furthermore, its wider impacts on society such as costs of treatment, costs of time that prevent children from attending school and their parents from attending work, has made it more serious [7, 9]. According to literature, ECC is a public health problem that was considered an epidemic condition in both industrialized and developing countries. The prevalence of this condition was reported between 1 and 12% among infants in developed countries and 52% with mean dmft = 1.9 among 3-year-old Iranian children [10–12]. In children under six years old, the influence of mothers’ and Primary Health Care Providers’ (PHCPs) attitudes, beliefs and practices are crucial. Babies who had mothers with decayed teeth were at increased risk of ECC [13]. WHO also stressed the urgent need for action to control this public health problem and suggested the population-based prevention of ECC using oral health educational interventions (such as avoidance of free sugars in complementary foods and drinks, promotion of breastfeeding, using finger brush or soft tooth brushing for children twice a day) targeting pregnant women, new mothers and PHCPs as well as interprofessional education with other health professions. Also, it is recommended that, ECC prevention measures must be planned at appropriate times, such as vaccination period and developing a training package for dental and non-dental staff to provide appropriate prevention and management of ECC is necessary [14].

According to evidences, it is significantly suggested that oral health promotion interventions within the first year of life are critical. Interventions that target mothers, both during pregnancy and in the first year after giving birth can effectively prevent ECC [14, 15]. However, insufficient well-designed methodologies for development of these interventions have led to unsatisfactory results and “despite the fact that many ECC prevention guidelines exist, their effectiveness in the final goal of obtaining a significant, long-term and homogeneous reduction of ECC incidence, is not proved” [16]. As mentioned above, previous studies in Iran reported rather high prevalence of ECC compared to other countries and also noted the need to take preventive measures [12, 17, 18]. Free maternal and child health services are provided in these Iran’s comprehensive health centers including pregnancy visits, pregnancy tests, baby growth supervision and vaccination by PHCPs. The national population coverage of child care (vaccination and growth monitoring of newborns) and maternal care (antenatal care coverage) was reported 98% [19] and 94.3% [20] in public health services, respectively. Using the potential of pre- and post-natal care provided to mothers and infants in these centers to perform ECC prevention interventions is a simple and cost-effective method. Educational interventions with appropriate design and content that are present by the forces present in the comprehensive health centers may be able to eliminate the weakness in the interventions that have been done so far. These poor reported results as well as uncertainty for the correct solution has prompted us to conduct the present study. Due to limited population in this area, it is not possible to have a fully scaled study but it is still important to test out different modified interventions in order to inform public health practice and so the aim of this study was to pilot and compare the effectiveness of four different, but overlapping community-based interventions for preventing ECC among children up to two years of age.

## Methods

### Study design and setting

**Fig. 1:**
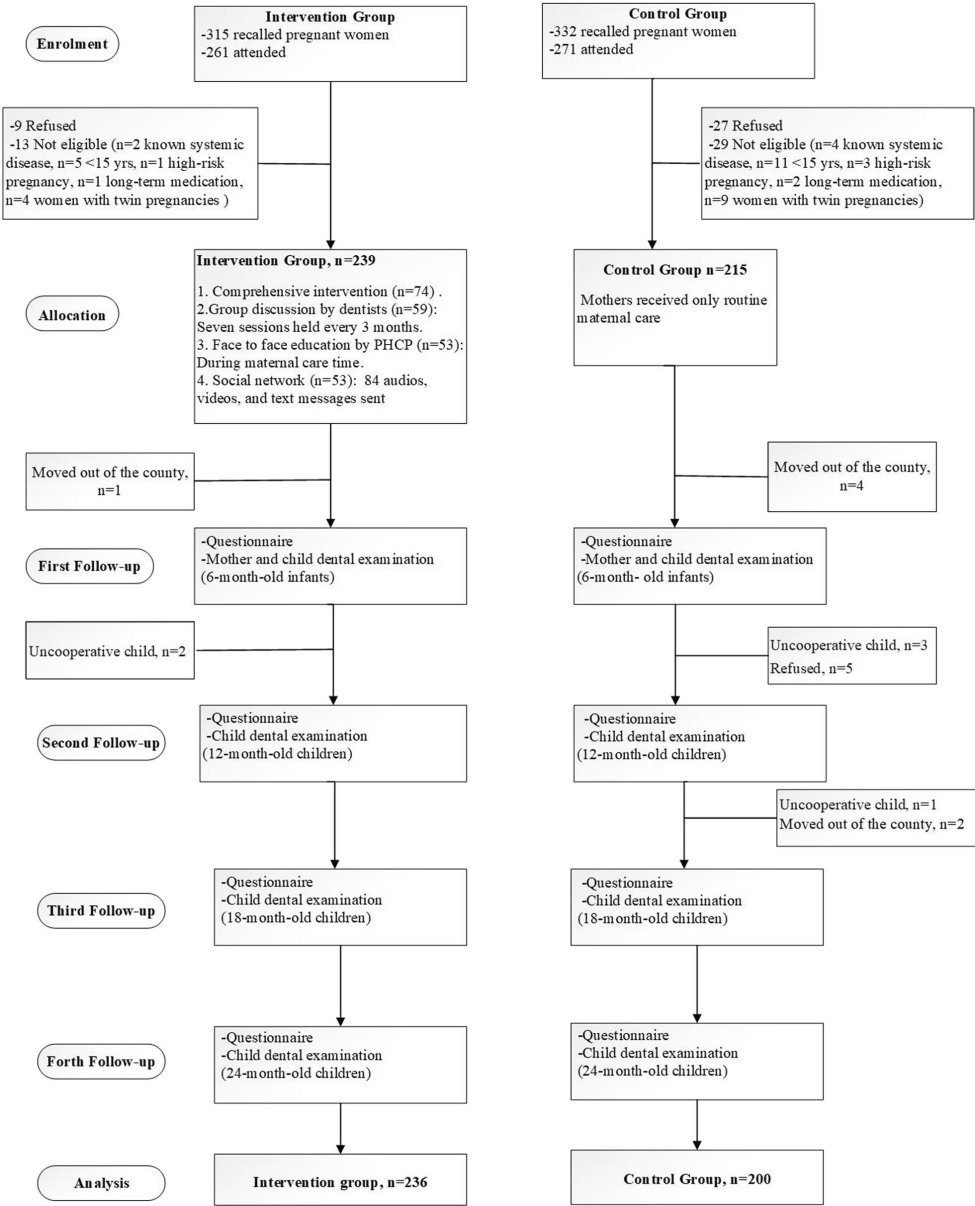
Flow chart of baseline to final follow-up study participants

This field trial study was designed to evaluate the effect of community-based interventions on dental caries prevention of newborns through 24-months follow-up. The target population comprised pregnant women who sought maternal care from all the 18 comprehensive health care centers in Pishva and Pakdasht and their infants from delivery up to two years old. Pishva and Pakdasht are partially deprived counties which are located next to each other in Varamin region, southern part of Tehran province, Iran, and are similar in terms of socioeconomic status. This trial involved four intervention and one control groups. After obtaining the consent, intervention group undertook oral health educational interventions. Mothers in control group received routine maternal and child care provided in public healthcare centers. The design of this study has been provided in flowchart, Fig. 1.

### Participants and subject recruitment

According to the Iranian National Oral Health Survey in 2004, 48% of Iranian three-years old children were caries free [21]. All registered pregnant women, in second/third trimester of pregnancy, with a pregnancy record (routine maternal care) in comprehensive health care centers in Pishva and Pakdasht (n = 647), were recalled by the PHCPs. Inclusion criteria were being in the second/third trimester of pregnancy and being over 15 years old. Also, women with known systemic disease, high-risk pregnancy, long-term medications, twin pregnancies, and uncooperative children were excluded. Moreover, those who did not participate in at least 3 face-to-face sessions or 5 sessions of group discussion with the dentist were excluded from the study. The samples were assigned to study groups, based on the information provided by local managers of the comprehensive health centers, population of participants in each center and creating maximum possible distance between participants in different groups. In all intervention and control groups, a questionnaire was administered, and dental examination of mothers was done at first follow-up when the infant was six months old. Recruitment of mothers started in July 2016 and lasted for eight months, and the newborns were followed up to 24 months old (February 2019).

### Intervention

Intervention programs were led by a team including a dental public health PhD candidate, a dental public health Associate Professor and some associates from Health Deputy of Shahid Beheshti University of Medical Sciences. After obtaining the informed consent, intervention group undertook oral health interventions during second and third trimester of pregnancy. These interventions reinforced later when their child reached 6, 12, and 18 months of age. Interventions were designed based on covering the existing gaps that obtained from a qualitative study [22], and included nutritional and behavioral messages that we extracted from nutritional and behavioral oral health recommendations from WHO and also American Academy of Pediatric Dentistry oral health guideline for infants and toddlers [23–25]. The educational content has been approved by Community Oral Health Department, Shahid Beheshti University of Medical Sciences. The common risk factor approach was used to design all interventions.

Nutritional messages consisted of mother and child healthy diet, encouraging daily intake of fruits and vegetables, restricting frequency of sweet consumption (e.g. juices, honey, chocolate, cookies, and sugar) in baby bottle. Behavioral messages included teaching the correct method of tooth brushing, encouraging mothers to brush their teeth twice a day, using fluoridate tooth paste, clarifying the importance of dental visits during pregnancy and lactating period for both mother and newborn, using chewing xylitol gum, avoiding any use of tobacco products, using finger tooth brush for the child, avoiding breastfeeding during the night and avoiding sweet bottle feeding at all times.

All eight health care centers in Pishva were divided into four group. Educational interventions in each group were performed using one of the following four methods. The content of health messages was similar in all groups.

A: Comprehensive intervention:

This type of intervention was performed on 74 mothers at Sadooghi and Kahnak health care centers and consisted of all the three mentioned interventions below.

B: Performing group discussion sessions by dentists:

Totally seven behavioral and nutritional educational sessions were performed every three months, from late pregnancy up to 18 months after delivery. Fifty-nine mothers participated in group sessions. Group sessions were held at the conference hall of Jalilabad and Tarand healthcare centers and they were facilitated by a trained dentist, a nutritionist and a midwife in each center. Lectures were delivered by the dentist to provide advices on the educational messages, group discussions (question and answer) about mothers and their newborns oral health behavioral and dietary issues. Main topics from previous sessions were asked and repeated in the next group session. At the first educational intervention, participants were given a pamphlet consisting of the given advices at the session, and a tube of fluoridated toothpaste accompanied with a tooth brush.

C: Face to face advice by PHCPs at routine maternal and child care visits:

All the PHCPs working in Pishva healthcare centers were trained during three workshops, focusing on the importance of pregnant women’s oral health and the essential role of PHCPs on improving mother and newborn’s oral health. Fifty-three mothers were educated by these trained PHCPs in Mohtadi and Jalilabad2 healthcare centers. These sessions were implemented at pregnancy, 1, 6, 12 and 18 months after delivery, when mothers attended health care centers for routine baby checkups (vaccination and growth supervision of newborns). PHCPs educated mothers on nutritional and behavioral issues using posters, pamphlets, dentate manikin head model and tooth brush, and finger brush.

D: Social networking applications:

A Dental Public Health PhD candidate created a channel on Telegram social network application and sent 84 behavioral (n = 41) and nutritional (n = 43) contents consisted of audios, videos, and text messages to 53 mothers enrolled in Mohajeri and Ardestani healthcare centers. Participants received messages every week starting from pregnancy period until 18 months after the delivery.

#### Control group

All the 215 mothers enrolled in 10 healthcare centers in Pakdasht were considered as control group and received routine maternal and child health and oral health services.

### Data collection and variables

In both intervention and control groups, a structured questionnaire was filled through face-to-face interviews and dental examination was performed in order to collect baseline data of mothers’ oral health status and also some demographic socioeconomic information including child gender, mother's age, level of mother's education, family income and family size at the first follow up when the newborns were 6 months old. Oral examination of children was done at 24 months after delivery and a questionnaire was filled to assess child’s dental care status (using finger brushing, daily sweet consumption and type of milk feeding) at 6, 12, 18, and 24 months of age. Two trained and calibrated dentists performed the oral examinations at the maternal care room in public health centers; using cotton rolls, battery-operated lights and mouth mirror to detect carious legions according to the World Health Organization (WHO) oral health surveys basic methods [26]. Mean inter-examiner agreement achieved in our activity was Kappa = 0.85. Both dentists were blinded to group allocation during oral examination. The dentist asked mothers to hold their children on their lap during the exam to explode the dental caries. Mothers were informed of oral examination results and, if needed, were advised to visit a public dental service for treatments. In the case of dental infections or emergencies, the participants were referred for immediate dental procedures to public dental services. After oral examination and interview, all the study participants were given a tube of fluoridated tooth paste, a tooth brush and a finger brush. The correct brushing method was demonstrated to all of the participants and they were advised to brush at least twice a day.

Outcome variable was the number of child’s decayed teeth (d) at the age of 24 months to calculate the rate of caries free participants. Being caries free was defined as any visible tooth in child’s mouth should not have any carious legion. To further reveal the clinical significance of changes in the rate of caries-free children in the intervention groups, the Numbers Needed to Treat (NNT) index was also calculated for each group. Intervention was the main explanatory variable. Demographic variables (child gender, mother's age), socio-economic status (SES) variables (level of mother's education, family income, family size), mother’s dental caries (D) at 6 months after delivery, and finally infant dental care behaviors variables (using finger brushing at 12 months, type of milk feeding at 6 months). Gestational age was recoded into “under 37 weeks” and “37 weeks and more”, and newborn weight was classified as < 2500 gr. and ≥ 2500 gr. The responses for sweet consumption were dichotomized into “no sweet” or “sweet consumption” at any time of a day. Information on the type of milk feeding was recoded into “only breast feeding” and “milk powder or both”.

### Statistical analysis

Statistical analysis and data preparation were done via IBM SPSS Statistics (version 19) software and STATA (version 14). For bivariate statistical analysis, Chi-square, Kruskal–Wallis, and Mann–Whitney U tests were used. Variables with *P* value ≤ 0.25 in bivariate analysis were entered to the multivariate analysis. The NNT index was calculated through its standard formula and according to the difference in the number of caries-free samples in each intervention group compared to the control group. To model being caries free, binary logistic regression models were used. Adjusted models were used to study the association between explanatory variables and the outcome variable. *P* value less than 0.05 was considered statistically significant. OR (95%CI) was reported for analysis of being caries free in binary logistic regression model regression. Generalized linear regression model was used for comparing being caries free at final follow-up among the five groups (four interventions and a control group) after adjusting for potential confounders.

### Ethical issues

This study was approved by the Committee of Ethics in Research Affairs of Dental School, Shahid Beheshti University of Medical sciences (code: IR.SBMU.DRC.REC.1397.003). Informed and written consent was taken from the mothers after providing detailed information on study objectives. Confidentiality of the participants was maintained throughout the study. One of the mothers at the baseline of study was under 16 years old whose consent form was signed by her father as her legal guardian according the local rules.

## Results

From the 647 registered women, 454 attended to an examination session, from which 436 were included in the analysis. Figure 1 shows the flow of the participants in intervention and control groups. During follow-ups, a total of six children (intervention, n = 2 and control, n = 4) were uncooperative and were excluded from the study.

Among the 436 examined children, with a mean age of 23.7 months (range: 22–25 months), 48.2% (n = 210) were male. Frequency of using finger toothbrush increased from the first follow-up to at the last follow-up in all intervention groups (Table 1).

**Table 1:** Participants’ distribution according to demographic, socioeconomic, dental care behaviors, and mothers’ dental caries variables in intervention and control groups (n = 436)

Characteristics	Comprehensive (n = 74)	Group discussion (n = 58)	Face to face by PHCPs (n = 52)	Social network (n = 52)	Control (n = 200)
	N (%)	N (%)	N (%)	N (%)	N (%)
*Demographic variables*					
Child gender					
Male	35 (47.30)	27 (46.60)	26 (50.00)	24 (46.20)	98 (49.00)
Female	39 (52.70)	31 (53.40)	26 (50.00)	28 (53.80)	102 (51.00)
Maternal age group					
15–25	22 (29.70)	21 (36.20)	16 (30.80)	19 (36.50)	58 (29.00)
25–35	45 (60.80)	29 (50.00)	31 (59.60)	27 (51.90)	112 (56.00)
35–44	7 (9.50)	8 (13.80)	5 (9.60)	6 (11.50)	30 (15.00)
*Socioeconomic variables*					
Maternal level of education					
Less than 12 years	42 (56.80)	28 (48.30)	31 (59.60)	28 (53.80)	116 (58.00)
12 years	27 (36.50)	28 (48.30)	18 (34.60)	18 (34.60)	71 (35.50)
More than 12 years	5 (6.80)	2 (3.40)	3 (5.80)	6 (11.50)	13 (6.50)
Family income					
Low	33 (44.60)	15 (25.90)	20 (38.50)	15 (28.80)	70 (35.00)
Middle	26 (35.10)	27 (46.60)	22 (42.30)	19 (36.50)	95 (47.50)
High	15 (20.30)	16 (27.60)	10 (19.20)	18 (34.60)	35 (17.50)
Family size					
3–4	50 (67.60)	40 (69)	36 (69.20)	40 (76.90)	132 (66.00)
5–6	24 (32.40)	18 (31)	16 (30.80)	12 (23.10)	68 (34.00)
*Dental care behaviors*					
Brushing habit (using finger brush)					
at 6 month*	48 (64.90)	24 (41.40)	35 (67.30)	19 (36.50)	23 (11.50)
at 12 month*	62 (83.80)	38 (65.50)	39 (75.00)	28 (53.80)	43 (21.50)
at 18 month*	70 (94.60)	48 (82.80)	48 (92.30)	37 (71.20)	51 (25.50)
at 24 month*	71 (95.90)	54 (93.10)	50 (96.20)	37 (71.20)	44 (22.00)
Type of (milk)feeding at 12 m*					
Breast milk	61 (82.40)	41 (70.70)	37 (71.20)	30 (57.70)	94 (47.00)
Milk powder and both of them	13 (17.60)	17 (29.30)	15 (28.80)	22 (42.30)	106 (53.00)
Child sweet consumption at 12 m*					
No sweet	63 (85.10)	40 (69.00)	34 (65.40)	27 (51.90)	71 (35.50)
Sugary bottle	11 (14.90)	18 (31.00)	18 (34.60)	25 (48.10)	129 (64.50)
*Mothers dental caries*					
D mother					
Without caries	2 (2.70)	5 (8.60)	3 (5.80)	3 (5.80)	8 (4.00)
With caries	72 (97.30)	53 (91.40)	49 (94.20)	49 (94.20)	192 (96.00)
Total	74 (100%)	58 (100%)	52 (100%)	52 (100%)	200 (100%)

*P value < 0.05

**Fig. 2:**
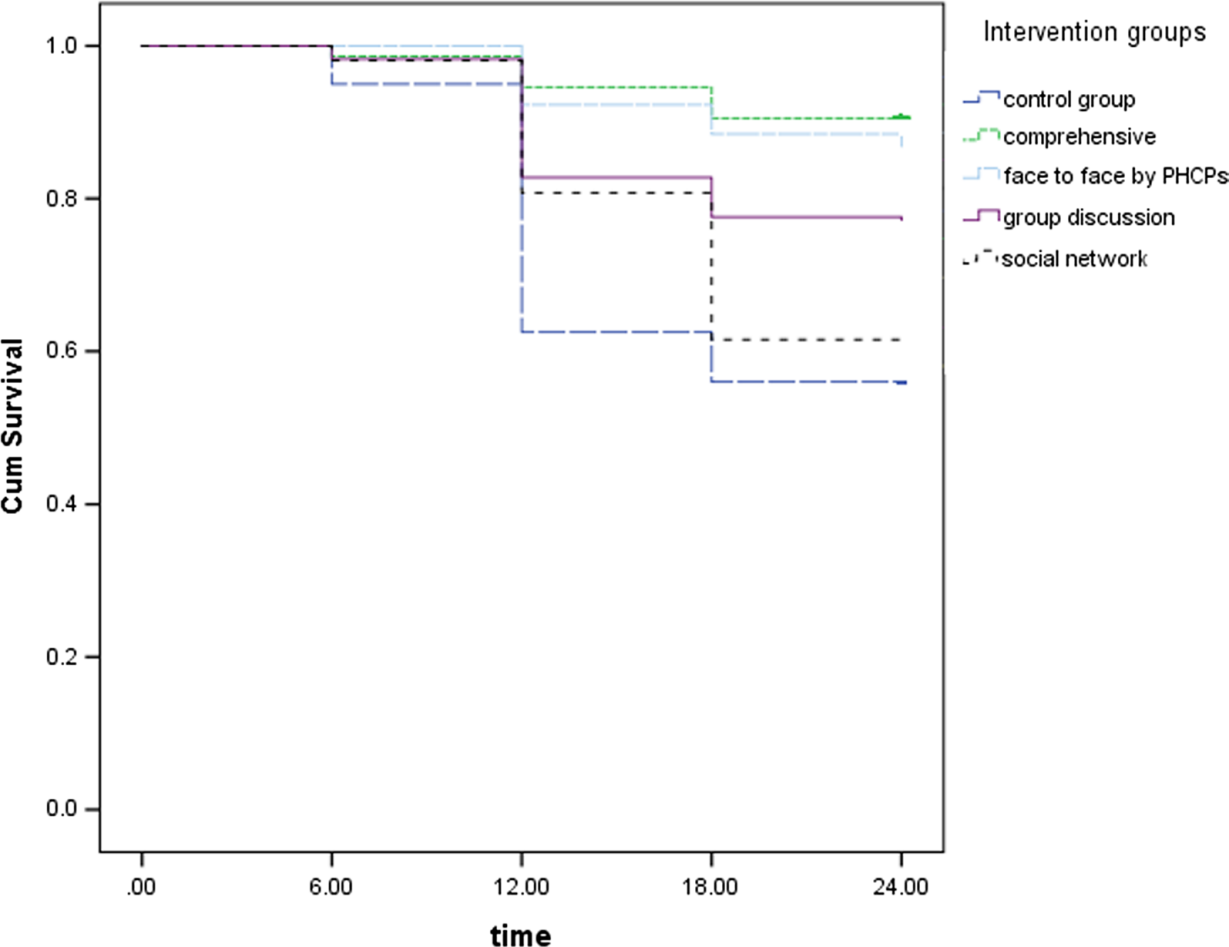
Kaplan–Meier survival curves for the incidence of first new caries in the studied groups

The mean (SD) of d at 24 months in intervention and control groups were 0.36 (0.93) and 1.61(2.61), respectively. Also, the mean (SD) d in A, B, C, D intervention groups were 0.11 (0.42), 0.55 (1.16), 0.17 (0.43), 0.71 (1.33), respectively. Figure 2 shows the estimated Kaplan–Meier survival curves that allow us to compare the experience of studied groups according to the incidence of new caries over time. By Log-rank test we found a significant difference between the survival experience of the studied five groups (*P* < 0.001). By making pairwise comparisons for the interventional groups with the control group by considering the Bonferroni correction, revealed that the comprehensive and face to face intervention groups showed the most significant differences with the control group (*P* < 0.001), Group discussion was also better than the control group (*P* = 0.048) and no significant difference was found for social network group compared with the control group (*P* > 0.05).

Considering caries free index, the numbers needed to treat (NNT) were 2.8, 5.5, 3.5, 27.7 for A, B, C, D interventions, respectively (Table 2).

**Table 2:** The numbers needed to treat (NNT) with (%95 CI) in four intervention groups

	Comprehensive	Group discussion	Face to face	Social network
NNT	2.8 (2.2, 3.8)	5.5 (3.2, 20.3)	3.5 (2.5, 6.0)	27.7 (5.4, infinity)

From the 236 children in intervention group, 186 (78.8%) were caries free at 24 months old, whereas in control group 112 out of 200 children (56%) had the same condition. In intervention group, percentage of caries free at the age of 24 months in A–D interventions were 68 (91.9%), 43 (74.1%), 44 (84.6%), 31 (59.6%) respectively. Also, children who had been fed on milk powder had more dental caries at 24 months of age compared to those who solely were breastfed (*P* value < 0.001) (Table 3).

**Table 3:** Relationship between demographic, socioeconomic, mothers’ dental caries variables and being caries free at 24 months

Variables	N (%) caries free at 24 months					
	Comprehensive (n = 74)	Group discussion (n = 58)	Face to face by PHCPs (n = 52)	Social network (n = 52)	Control (n = 200)	Total
*Demographic variables*						
Child gender						
Male	31 (88.60)	20 (74.10)	21 (80.80)	15 (62.50)	55 (56.10)	142 (67.60)
Female	37 (94.90)	23 (74.20)	23 (88.50)	16 (57.10)	57 (55.90)	156 (69.00)
*P* value						0.75 ^b^
Maternal age group						
15–25	20 (90.90)	17(81.00)	15 (93.80)	11 (57.90)	36 (62.10)	99 (72.80)
25–35	41(55.40)	21(72.40)	25 (80.60)	16 (59.30)	61 (54.50)	164 (67.20)
35–44	7 (100)	5 (62.50)	4 (80)	4 (66.70)	15 (50.00)	35 (62.50)
*P* value						0.32^a^
*Socioeconomic variable*						
Maternal level of education						
Less than 12 years	39 (92.90)	23 (82.10)	25 (80.60)	16 (57.10)	63 (54.30)	166 (67.80)
12 years	24 (88.90)	19 (67.90)	17 (94.40%)	10 (55.60)	42 (59.20)	112 (69.10)
More than 12 years	5 (100)	1 (50)	2 (66.70%)	5 (83.30)	7 (53.80)	20 (69.00)
*P* value						0.95^a^
Family income						
Low	31 (93.90)	10 (66.70)	16 (80.00)	8 (53.30)	37 (52.90)	102 (66.70)
Middle	23 (88.50)	19 (70.40)	20 (90.90)	10 (52.60)	55 (57.90)	127 (67.20)
High	14 (93.30)	14 (87.50)	8 (80.00)	13 (72.20)	20 (57.10)	69 (73.40)
*P* value						0.49^a^
Family size						
3–4	46 (92.00)	29 (72.50)	31 (86.10)	24 (60.00)	80 (60.6%)	210 (70.50)
5–6	22 (91.70)	14 (77.80)	13 (81.30)	7 (58.30)	32 (47.10)	88 (63.80)
*P* value						0.16 ^b^
Type of(milk) feeding at 12 m						
Only breast milk	61 (100.00)	39 (95.10)	36 (97.30)	26 (86.70)	83 (88.30)	245 (93.20)
Milk powder and both of them	7 (53.80)	4 (23.50)	8 (53.30)	5 (22.70)	29 (27.40)	53 (30.60)
*P* value						** <0.001**^b^
Mothers dental caries						
D mother	1 (50.00)	5 (100.00)	3 (100.00)	3 (100.00)	4 (50.00)	16 (76.20)
Without caries	67 (93.10)	38 (71.70)	41 (83.70)	28 (57.10)	108 (56.30)	282 (68.00)
With caries						
*P* value						0.42^b^
Total	68 (91.90)	43 (74.10)	44 (84.60)	31 (59.60)	112 (56.00)	298 (68.30)

^a^Krusal–Wallis test

^b^Mann–Whitney U-test

Bold: relationship significant at 5% level

According to Table 4, all the four intervention groups, except D (social network), resulted in more chance of being caries free when compared to control group. Children in intervention group A showed the lowest rate of dental caries compared to control group [OR = 8.67 (95%CI 3.59; 20.94)]. After adjustment for covariates, intervention group A had still significantly less dental caries [OR = 4.54 (95% CI 1.58;12.94)] compared to control group. Also, in the adjusted model, children being fed with milk powder had thirty-seven times more dental caries compared to those who were breastfed after controlling for the type of interventions and other covariates [OR = 37.62 (95% CI 19.39;73.00)].

**Table 4:** Association between demographic and dental care behaviors with being caries free at 24- months-old (n = 436)

d at 24 month	OR(95% CI)
*Model 1*	
Intervention	
Control	**1**
Comprehensive	**8.67 (3.59, 20.94)**
PHCPs	**4.19 (1.87, 9.38)**
Group discussion	**2.26 (1.18, 4.35)**
Social network	1.10 (0.59, 2.06)
Child gender	
Male	1
Female	1.04 (0.68, 159)
Mother’s age	0.96 (0.93, 1.00)
*Model 2*	
Intervention	
Control	**1**
Comprehensive	**8.79 (3.63, 21.26)**
PHCPs	**4.22 (1.88, 9.45)**
Group discussion	**2.25 (1.17, 4.34)**
Social network	1.09 (058, 2.04)
Child gender	
Male	1
Female	1.02 (0.66, 1.57)
Mother’s age	0.98 (0.93, 1.03)
Family size	0.82 (0.60, 1.13)
*Model 3*	
Intervention	
Control	**1**
Comprehensive	**8.61 (3.54, 20.92)**
PHCPs	**4.18 (1.86, 9.37)**
Group discussion	**2.26 (1.17, 4.35)**
Social network	1.08 (057, 2.02)
Child gender	
Male	1
Female	1.02 (0.66, 1.57)
Mother’s age	0.98 (0.94, 1.04)
Family size	0.82 (0.59, 1.13)
D mother at 6 m	1.01 (0.96, 1.07)
*Model 4*	
Intervention	
Control	**1**
Comprehensive	**4.54 (1.58, 12.94)**
PHCPs	**3.16 (1.16, 8.57)**
Group discussion	1.22 (0.50, 3.00)
Social network	0.63 (0.27, 1.51)
Child gender	
Male	1
Female	1.10 (0.62, 1.94)
Mother’s age	0.97 (0.90, 1.03)
Family size	0.60 (0.39, 0.92)
D mother at 6 m	1.01 (0.94, 1.09)
Type of milk feeding	
Powder and both of them	1
Breast feeding Milk	**37.62 (19.39, 73.00)**

Generalized linear regression model

*OR* odds ratio, *CI* 95% confidence interval

Model 1: adjusted for Intervention, child gender and mother’s age

Model 2: adjusted for Intervention, child gender, mother’s age and family size

Model 3: adjusted for Intervention, child gender, mother’s age, family size and D of mother

Model 4: adjusted for Intervention, child gender, mother’s age, family size, D of mother and type of milk feeding

Bold: relationship significant at 5% level

## Discussion

We conducted this study to evaluate the effectiveness of particular educational/behavioral interventions aimed to prevent dental caries in children. Regarding the new FDI World Dental Federation’s recommendation, integrating oral health into the general health, we used trained and calibrated PHCPs to implement interventions along with dentists in order to achieve better results [27]. Moreover, our design took advantage of feasible, cost effective and applicable interventions which are necessary requirements for any health promotion actions especially in deprived areas like this study’s field. The retention rate in our study was very high (96%) due to lack of migration in this country and also the tendency for reside in central parts of Iran especially around Tehran [28]. Also, our interventions were time friendly and easy to access which resulted in more retention rate of participants in study.

Results of the present study reported no statistically significant relationship between maternal age group, mothers' level of education, family income and children dental caries. Our results are in line with previous study which reported that there was no significant correlation between SES and the severity of ECC [29]. On the contrary, some other studies showed that, there was an association between high prevalence of ECC and low family incomes [30, 31]. However, it may be because more than 90% of mothers in our study had educational level less than 12 years. Mothers with low level of education are rarely exposed to oral health information during primary education [32], and are less likely to be sensitive to the importance of their newborn’s oral health care and healthy nutrition. Also, present study aimed at some deprived areas, and Similar to mothers' educational level, majority of participants had moderate and low family income and this lack of diversity may made our comparisons not statistically significant.

After the follow-ups, in total about one third (31.70%) of children, aged 24-month-old, were diagnosed with ECC. The prevalence of ECC has more than doubled in control group compared to intervention group. Even after adjustment for demographic, socioeconomic, child dental care behaviors and mother’s dental caries variables, intervention group, still had significantly less dental caries compared to control group. The results of our previous study that were carried out in 2015 on three-year-old children in the same area showed that 56.60% of children had ECC [33]. Also, Toutouni et al., in 2015, in a study on the prevalence of ECC among 2 -3-year-old children in Iran using International Caries Detection and Assessment System (ICDAS), reported very high caries prevalence, with about 60% of samples displayed pitted caries [34]. However, this highly divergent results may be due to the different nature of indexes used in each study. Another study in Philippines reported that 59% of 2 years old children had ECC [35]. Comparison between reported prevalence of ECC in our intervention group and control group and also other local or worldwide results indicates the noticeable effectiveness of our interventions in ECC prevention. This finding was consistent with the results of Azevedo study that conducted educational intervention on mothers of 1 years old children. She found out that the children in the intervention group had significantly fewer carious lesions than those in the control group and the odds of dental caries were 80% greater in the control group compared to intervention group, after adjustment for the confounding effect of the number of teeth and child’s age [36].

Our study interventions consisted of comprehensive (A), face to face education by PHCPs (B), group discussion by dentists (C), and social network (D) interventions. The least mean value of decayed teeth (d) and also NNT indexes among all intervention and control groups belonged to comprehensive (both of them were nearly ten times less than the control group) and then PHCPs’ intervention groups. The NNT value gave us a useful tool for the interpretation of the interventions from a clinical and cost-effective point of view. It shows the high effectiveness of the comprehensive intervention compared to others, especially regarding prevention of dental caries. Mohebbi et al. reported that, the NNT was nearly half for group 1 (educational pamphlet, 5 min instructions, two recall phone calls at 2-month intervals) compared to group 2 (educational pamphlet with a comment that it would be useful to read) [37].

Based on our findings, the frequency of children’s daily oral health cleaning habit (using finger tooth brushing) at 24-month of age was about 30% higher in intervention groups compared to control group. Our findings are consistent with the results of the study conducted by Basir et al. in Iran [38] and also an Indian study aimed women who had 6 -18 months old children [39]. All these findings revealed that oral health educational intervention improved the performance of women who brush their young children’s teeth as one of the most effective methods to prevent ECC.

Among four methods we used in this study, intervention A was the most effective one to reduce ECC in our study participants. This may be because of using various educational methods and different messengers (dentists, PHCPs, social media) with more repetition in order to convey health messages to participants. Also, it seems that some of these particular messengers may have been more effective on some participants; it means some mothers based on their personal mentality, may be much more impressed if they were advised from a dentist than a midwife and vice versa. In line with results of our study, an Australian study found that oral health promotion interventions based on repeated rounds of anticipatory guidance including mail and telephone consultation was successful in reducing the incidence of ECC among the young children [40]. In addition, Mohebbi et al. revealed that children in the group whose mothers received verbal explanation on oral health information (pamphlet) with extra reminders (recall phone calls) had lower dental caries compared to intervention group without reminder and control group [37]. Also, Winter et al. found that interdisciplinary prevention (by gynecologists, midwives, pediatricians, dentists, municipal social services, and the public health office) program had an effective influence on preventing ECC [41].

Following intervention A, intervention B was considerably superior in controlling the amount of caries over the follow-up period. Mothers pay a lot of attention to their baby's general health during pregnancy and lactation. Also, nearly 70% of mothers indicated that they have learned about feeding methods from PHCPs. Considering these results, it seems oral health messages sent by PHCPs, are significantly more effective because they pay close attention to the general health of mothers and children, as well as the growth of the children. Furthermore, pregnant and lactating mothers frequently refer to PHCPs for prenatal care, child development, and vaccination of their children, but they may not visit a dentist meanwhile. Edelstein et al. reported that prenatal and immediate postnatal interventions are effective on ECC prevention when performed by community health workers [42]. Based on these results, we concluded that educated PHCPs in healthcare centers are uniquely positioned to provide feasible and cost-effective measures like ECC assessment, preventive and educational interventions, referrals and finally it is also in line with the new FDI recommendation about oral and general health integration.

Intervention D alone has not been effective in our study compared to other methods. On the other hand, an Iranian study published in 2020 reported that oral health intervention delivered by social media (Telegram) can improve oral hygiene behavior such as tooth brushing and oral health outcomes among Iranian adolescent students and their mothers in short and long-term periods [43]. These contradictory results about using social media as a mean to improving oral heath may be related to the fact that lactating mothers are not interactive enough in social media and pay more attention to face-to-face meetings by dentists and PHCPs. also, after giving birth during lactating periods, they did not pay enough attention or had not enough time to check and read health-related messages from social networks and peruse recommendations thoroughly. Although this method found not to be effective in our study, it may be recommended because of low cost and availability.

Another finding of our study was that, children who solely went through breastfeeding had less dental caries at 24 months of age compared to those who had been fed on milk powder.

Breastfeeding is the best natural way of nourishing infants and it is necessary for the healthy development of the child. Breast milk has buffer capacity that eventually may prevent dental caries as well. Antibacterial quality of breast milk can interfere with activity of microorganisms that cause dental caries [44]. A study in Brazil revealed that children who were never breast‐fed or were breast‐fed beyond the 24 months of age showed a higher prevalence of extensive caries compare to children who were breastfed only [45]. Also, a meta-analysis study on breastfeeding and ECC concluded that breastfeeding may protect children from ECC [46].

## Limitations

We should acknowledge that all the findings might not be generalized to other countries because of the distinctive culture and structure of the healthcare system in the Islamic Republic of Iran. However, it may be appropriate to be considered for similar deprived areas in Iran. Unfortunately, due to the limited population of the two studied cities, the study team did not have enough samples to fulfill the participants’ estimated size and the study is underpowered (CI is broad due to this issue). This may be the reason for not seeing a significant relationship between the interventions and the change in indicators related to oral health in the social media group.

This should be mentioned that for those who did not participate in the study, the Comprehensive Health Centers did not provide patient information to the study team due to confidentiality.

Also, random assignment was not applicable in our study due to geographical proximity of place of residence in each area that increased chance of data transmission between intervention groups. Some women were not reachable for social network intervention because they did not have smart phones or did not use social applications.

## Conclusion

While the sample recruitment limitations does create some issues in comparability of groups and generalizability of findings, this series of pilot interventions showed that the most effective way to prevent ECCs would be implementing comprehensive oral health intervention as a part of the healthcare provided to pregnant and lactating women by PHCPs and dentists in healthcare centers. Further studies are needed to validate this type of intervention.

## Data Availability

The data that support the findings of this study are available from the corresponding author upon reasonable request.

## Abbreviations

CI: Confidence intervals
d: Decayed teeth
ECC: Early childhood caries
NNT: Numbers needed to treat
PHCPs: Primary health care providers’
SES: Socio-economic status
OR: Odds ratio
SD: Standard deviation
WHO: World Health Organization
